# On the Treatment (by Counter-Irritation) of Organic Diseases of the Heart

**Published:** 1830-10-01

**Authors:** 


### On the Treatment of Organic Diseases of the Heart. By Baron Larrey.

It is scarcely less distressing to the medical practitioner to witness the slow ravages of an indomitable organic disease, than for the patient to bear them. The venerable Baron Larrey has dedicated a chapter in his recent work, to the treatment of enlargements of the heart, by means of counter-irritation, especially by moxa, which we deem it necessary to notice in this Journal.

The more, he says, we reflect on organic alterations in the heart, the more we shall be convinced that they, like other changes of structure, result from a morbid irritation going on in the tissues of the heart, and disturbing its functions. Contrary to the opinion of Corvisart, Baron Larrey is of opinion that we cannot remove hypertrophy of the heart by the most rigorous system of depletion—and in this sentiment we coincide. Counter-irritation, however, promises more advantages, and the Baron assures us that, for more than thirty years past, he has ascertained the beneficial effects of this counter-irritation on a large scale. The Baron has a theory in his head that the primary and essential cause of these organic diseases consists in the presence of "a morbific principle, whether syphilitic, scrofulous, herpetic, or otherwise, which fixes itself on the dense tissues of the heart and arteries." This doctrine, especially when taken in connexion with the remedy, bears some analogy to that of St. John Long—but the veteran Frenchman does not attempt to extract money from the pockets and quicksilver from the brains of his patients! He fairly states the mode and means of counter-irritation—and is respected by his professional brethren—while the English charlatan applies AQUA FORTIS to the backs of his patients with as much *sang froid* as he formerly applied oil or varnish to the canvas of his paintings!

Baron Larrey thinks that the position of body necessitated by certain trades and professions,—the compression of the chest and abdomen by certain articles of dress—the violent exertion of the voice by singers, &c.—long-continued grief—onanism, and other causes are very efficient in the production of cardiac and arterial aneurisms. We shall not enter into the consideration of the symptoms and diagnosis of enlargements of the heart, whether active or passive, since we are constantly referring to such subjects. In respect to the treatment, Baron L. observes that all kinds of depletion have been recommended by authors, for the active hypertrophy; while the passive dilatation has been treated by diuretics and other remedies that, in his experience, have done more harm than good.

The Baron therefore lays down two indications to pursue, whether the enlargement be active or passive:—first, to counteract or destroy the primary specific cause (as syphilis, scrofula, &c.) if it can be recognized, by specific remedies—the second indication is to draw off irritation from the interior to exterior. Whether the primary cause be syphilitic, scrofulous, rheumatic, or herpetic, the Baron avers that the mercurial treatment is almost always beneficial, especially when combined with proper auxiliaries and counter-irritation. In the active hypertrophy, he uses local depletion as one of the auxiliaries—and after this remedy has been employed, he has recourse to the moxa, as the best method of producing counter-irritation. Cold, in the form of ice, is another favourite remedy of the Baron. It should be applied to the region of the heart. Next he commences the application of moxas in the track of the intercostal nerves behind the left hypochondrium, and gradually coming forward to the cardiac region anteriorly. The moxas here produced the best effects. A favourite prescription of the Baron's we shall here introduce.

℞. Oxymur. Hydrarg.

Muriatis Ammoniæ,

Opii, āā. gr. v.

Aquæ destillat. ℔j.

Misce ft. solutio.

A dessert spoonful of this solution is the dose prescribed, but whether oftener than once a day is not mentioned. The diet, of course, in active aneurism of the heart, is recommended to be very sparing. Moral and physical quietude is necessary; but, alas! how seldom can these be obtained! The Baron next proceeds to a detail of cases: as these are authentic facts, we shall make no apology for giving a succinct account of them.

*Case* 1. This was a young woman, 26 years of age, who presented the usual symptoms of hypertrophy of the heart. This organ beat over a large space—the impulsion was considerable—she could not bear pressure on the cardiac region—the pulse was hard and concentrated—she had great pain in the loins and region of the heart—jugular veins distended—lips violet—breathing difficult—aphonia complete. These symptoms had come on after a bad labour, succeeded by great uterine discharge (leucorrhœa) which was suddenly suppressed by saturnine injections, immediately after which, the cardiac affection supervened. These phenomena led Baron Larrey to conclude that *passive aneurism* of the heart existed—though we cannot see clearly the data on which the venerable author founded his assumption.

The oxymuriate solution, before alluded to, was prescribed for this patient, and cupping-glasses were successively applied to the loins and lumbar region generally, after which the moxa was used to various parts of the back, and round along the ribs to the region of the heart. The most rigorous regimen was enjoined, and the counter-irritation was long continued. The Baron confided his patient to the care of a pupil and went off on the Russian campaign. On his return, in 1815, he was agreeably surprised by a visit, at the "Hôpital de la Garde," from the patient (who was a washerwoman) in perfect health, and quite embonpoint.

With all due veneration for the experienced Baron, we doubt the existence of either passive or active dilatation of the heart in the above case. We have seen so many instances of inordinate action of the heart in females, after, or during uterine discharges, imitating organic disease, and deceiving ourselves and many of our professional brethren, that we have long ago learnt to distrust the common symptoms, and even auscultation in such cases. We strongly suspect that the foregoing case was one of these; but we are far from denying that the remedies were advantageous.

Case 2. A female, aged 27 years, had complained for about a year, of pain and violent palpitation in the region of the heart—symptoms which she attributed (and probably with some truth) to profound distress of mind which she had experienced, together with the sudden suppression of a fluor albus, to which she had been subject for many years. The Baron recognized "*all the signs of passive aneurism of the second degree of intensity.*" "Des battemens précipités, occupant un grand espace, se faisaient sentir sous les fausses côtes du côté droit, à l'epigastre, comme a tout le coté gauche de la poitrine." These palpitations (says Baron Larrey) were accompanied by pains in the back, cephalalgia, dry cough, dyspnœa, greenish expectoration, &c.* On placing the hand on the left side of the chest, it was strongly elevated by each pulsation of the ventricles, and conveyed a sense of heat to the observer. The patient was deprived of sleep, and was excessively depressed in mind. The treatment was commenced by a large bleeding from the jugular, after which cupping-glasses were applied to the loins. To these succeeded the moxas, two at a time, behind the left hypochondrium. The amelioration which the patient speedily experienced, encouraged her to persevere for the space of 15 or 16 months, during which 19 applications of the moxa were made. Ice was applied to the præcordial region for the first month, and the mercurial solution before mentioned was taken internally, with cooling and abstemious diet. Under this plan, all the symptoms of cardiac aneurism disappeared, and the heart regained its regular and natural action.

Case 3. Jean Baptiste, a lieutenant in the fifth regiment of Guards, aged 39 years, and of robust constitution, had made a great number of campaigns in various parts of Europe. He was suddenly seized, towards the close of the year 1824, with acute pain in the region of the heart, accompanied by audible palpitation, cephalalgia, vertigo, and occasional syncope, especially when exerting himself in military manœuvres. He also complained of a sense of oppression and much heat in the left side of the chest. He informed Baron Larrey that he had felt more or less of these symptoms ever since a third attack of syphilis, in which nodes on the shins and other constitutional symptoms had shewn themselves. He had several times had gonorrhœa, which

* It is astonishing to see such palpable inconsistencies as we often observe in the statements of the most eminent French practitioners. In the very same line of the same sentence, Baron Larrey asserts that the cough was *dry*, and accompanied by a *greenish expectoration!*

was treated by astringent injections. He entered the hospital in the beginning of August, 1825; and, on examination, the heart was pronounced to be in a state of active aneurism or hypertrophy. The left side of the chest was considerably larger than the right, and the ribs were separated more than on the opposite side. The pulsation of the heart was obscure, but accompanied by bruit—pulse full, hard, and vibrating, 100 in the minute. The left side of the thorax was hotter than the other—voice hoarse—great irritability, amounting to irascibility.

The jugular vein was opened—and, a few days afterwards, venesection was performed in the arm. Cupping-glasses were also applied to the back and loins, with scarification. A seton was inserted in the left side of the chest, and when suppuration was established, the moxas were commenced. The first two were applied under the left scapula, near the spine; and others were successively applied to the same place, as well as to the epigastrium. Thirteen applications were made in all. The actual cautery, in a gentle degree, was also employed a few times. The antisyphilitic solution was, of course, administered internally, with abstemious diet.

Ice was applied to the region of the heart, but he could not bear it. By perseverance during the space of eight months on this plan, the aneurismal symptoms disappeared—the left side of the chest was reduced *below* the size of the other. In short, he perfectly recovered his health, and was examined many times by physicians of eminence since his departure from the hospital.

*Remarks.* We suspect that, in this case, there was chronic inflammation of the pleura, with more or less effusion into the left side of the chest. The inflammation may also have affected the pericardium. We can in no other way account for the enlargement of the left side of the thorax first, and its ultimately becoming smaller than the other. The treatment, however, was good, whatever name we give the disease.

*Case* 4. Pierre D. a Swiss valet, presented himself to the Baron with all the symptoms of passive aneurism of the heart and incipient phthisis. The pulsations of the heart were inordinate, and over a large space—that side of the chest was of higher temperature than the other. Oppression of breathing, aphonia, habitual cough, with mucous expectoration, deep-seated pains in the loins and in the region of the heart were also present. Palpitation and bruit were heard when the ear was applied to the chest. The treatment was nearly the same as that which has been described in the other cases—namely, by local bleeding to a certain extent of reduction, when the moxas were applied for the space of 15 or 16 months, when a complete cure was effected.

We shall analyze but one other case, and then close this paper.

*Case* 5. Early in March, 1826, Alexandrine W——, aged 25 years, was brought to Baron Larrey. She was emaciated, pale, eyes hollow, breathing short and laborious, cough dry, with oppression, voice hoarse, mammæ wasted, and the nipples covered with an ill-conditioned ulceration. To the right of the centre of the sternum arose a prominence, the size of a small apple, under which were heard the pulsations of the heart, accompanied by a *bruit de soufflet*, synchronous with the pulse. When the ear was applied to this part the undulation appeared so close, that Baron Larrey conceived there was an aneurismal dilatation of the aorta, near its curvature, of considerable magnitude. The right carotid artery was much dilated up to its bifurcation. So were the subclavian and axillary of that side. The heart appeared, from auscultation, greatly enlarged, with thickening of the left ventricular parietes in particular. The least degree of exercise, or the slightest mental excitement brought on violent palpitation, oppression of the breath, and tendency to syncope—generally followed by hæmoptysis, with a pulse from 140 to 150 in the minute. We need not detail the various distressing symptoms which accompanied such a state of things. Suffice it to say, that they were aggravated by the indiscretion of a medical practitioner, who coolly informed this young lady that she laboured under a mortal malady!

When she came under Baron Larrey's care, in company with M. Dumeril, she informed them that, in 1820, she lost her mother, an elder sister, and a brother of aneurism of the heart, accompanied by phthisis pulmonalis. Soon after this series of afflictions, she began to experience the symptoms of her present complaint, in the form of violent palpitations and spittings of blood, with irregularity and even suppression of the menses. She had been bled several times, and was put on the most abstemious diet; by which she was relieved, with the help of assurances that her disease was not so bad as her other physician had predicted. But she relapsed into a state of great suffering, and it was at this epoch she came under the observation of our author. He concluded that an active aneurism of the heart existed in a very high degree, together with an aneurismal dilatation of the arch of the aorta, an enlargement of the innominata, and its two principal branches, the subclavian and carotid. Although no hope of cure was entertained by Larrey and Dumeril, they tried the effects of art. Cupping-glasses were applied in various places, and then the moxas. Ice was put to the region of the heart, and the mercurial solution, in small doses, was exhibited internally. A surprising benefit was experienced by these measures. The aneurismal tumours diminished—and the size of the heart appeared to be greatly lessened. Forty applications of the moxa were made, and the pulsating tumour of the sternum nearly disappeared. This interesting young lady gradually recovered health, and all the functions were restored almost to a state of perfect integrity. For the truth of these assertions, Baron Larrey appeals to the testimony of M. Dumeril. She became embonboint, and continued in apparently perfect health for several months, when, after many errors of diet, regimen, and exercise, she was seized, in the Spring of 1827, with an inflammatory catarrhal fever, suffocating cough, spitting of blood—but without any return of the aneurismal phenomena. Venesection and other means were employed, but phthisical symptoms set in—purulent expectoration became established—and hectic fever, with diarrhœa, completed the catastrophe.

*Dissection.* A considerable quantity of air was found in the *right* thoracic cavity, and the lung of that side was compressed to one-half its natural size. It was studded with tubercles, some of which were softened down. The calibre of the arch of the aorta was reduced below its medium size; but the ribs were still elevated at the point where it had been formerly dilated.* The *left* lung was atrophied, especially at its inferior part; but the upper lobe was sound and crepitous. The pericardium was thickened anteriorly—the heart was void of blood—*and smaller than that of a child.* The calibre of the aorta ascendens was less than natural. Its coats were thickened. The same might be said of the principal branches which arose from the aorta. The liver was prodigiously enlarged, and occupied half the right side of the abdomen. It had elevated the false ribs of that side, and also the diaphragm. Its substance was not altered. There was no other disease.

*Remarks.* There are some parts of the above case so marvellous, that, were we not assured of the worthy author's fidelity, we should suppose them the statements of a Baron Munchaussen rather than those of a Baron Larrey.

"L'exubérance (says he) formé par la troisième côte et son cartilage très-amenci presentait en dedans un courbure ou concavité proportioné a la saillie exterieure, et sous laquelle la crosse de l'aorte paraissait s'etre logée, lorsqu'elle était anevrismée, tandis que cette artère, réduite au-dessous du calibre de son etait normal, etait eloignée des parois de cette courbure de plus d'un pouce." This reduction of the aneu-

* This assertion staggers us a little; but as it is positively made, we dare not deny its truth.

rismal arch, when taken in connexion with the diminished size of the heart, is truly surprising, and, if correct, impresses us with a high opinion of the value of long-continued counter-irritation, in cases of organic disease within the chest. We think these cases deserve the attention of practitioners, when they have to deal with those dreadful enlargements of the heart, now so common in this and in other countries.

